# The Preparation of Cavities for Contour Fillings

**Published:** 1900-08-15

**Authors:** Wm. E. Harper


Dr. Wm. E. Harper, of Chicago, then read a paper on "The Preparation of Cavities for Contour Fillings."

### THE PREPARATION OF CAVITIES FOR CONTOUR FILLINGS.

BY WM. E. HARPER, D.D.S.

Of the failures in dental practice it has been my experience to observe that none were more frequent and humiliating than the gold fillings of mesio and disto-incisal cavities in the centrals and laterals; and this, too, after years of experience, painstaking and conscientious effort, with a liberal dental education as a foundation.

The triangular outline of the proximal surfaces with the narrow labio-lingual diameter in the incisal third of the crown, at which point the incisal and lingual force is continually exerted, is the chief cause of these failures, because of the small amount of dentin in which we may cut retention at this point.

To most effectually prevent the movement of a filling, the resistance should be cut flat in all directions and should be at right angles to the direction in which the filling is disposed to move; this applies equally to the floor or seat, which resists the incisal force in these cavities, and the retention, which is intended to resist the tipping either to the labial, mesial or distal.

In the cavity I present for your consideration (a mesio-incisal) the dentin of the cervical wall constitutes the seat and is made flat in all directions and as large as the enamel, which must be supported by dentin, and the pulp will permit.

In this cavity the resistance to force from any and all directions is more extensive and more effectually located and shaped, with the least possible sacrifice of tooth substance, than in any other method of preparation with which I am familiar.

With the proper instruments it is exceeding simple to prepare, and requires less of time and filling material than the step cavity and offers greater security to the filling.

To carry out this method of preparation it is imperative that instruments of the exact size and form as those indicated be used; otherwise the essential detail, upon which the greater security of the filling is dependent, will be in part lacking.

It is in these details only that the cavity differs from those prepared by many operators of the present time.

For retention, operators generally make a substantial undercut at the cervical under the curves of the labio and linguo cervical enamel wall. This is continued as a groove toward the incisal along the axial wall to the labial and lingual, and is made with a round or inverted cone bur, which, if cut to a depth of half the diameter of the head of the bur, would leave a half round groove.

This groove is a very important factor in the cavity I present. In the modification I suggest the depth remains about the same but differs in form, being made square with instruments mentioned below.

If each of these grooves be filled solidly with gold, is there any question as to which will most effectually resist displacement? To my mind a half round groove offers no security, while a square groove of less depth is retentive under force from all directions. With this conclusion I angle or square out all grooves, undercuts and junctions of different walls.

A square retainer, angle or groove can be more perfectly filled than a pit, groove or seat with a concave or half rounded bottom for a foundation on which to condense the gold.

I shall not attempt to argue the matter with those who question this statement, as it is best demonstrated. I would ask that you fill with gold a square groove with such instruments or pluggers as it is advisable to use for filling teeth, then cut through the filling and matrix and examine under the microscope. The result will speak for itself.

In cavities of this class it is generally necessary to secure separation for restoration of contour and to permit finishing. With the rubber dam in position we proceed to cleave down all unsupported enamel, including the weak incisal angle. This is done with a sharp, medium sized chisel fifteen-tenths of a millimeter wide. The labial and lingual margins in their incisal half should be cut to the nearest developmental groove, as these are weak lines of enamel and should be included in the cavity. The cervical or gingival margin should be approached by a long, smooth curve, and in its middle two-thirds should be cut straight labio-lingually. If the interproximate gingivæ be normal, this margin should be located beneath it for protection. If there has been considerable recession this extension may be inadvisable, in which case the cervical margin should be located at a wide portion of the interproximate space but well away from the gum margin and the contact point. With a well-contoured filling in such a position, the saliva and ordinary prophylactic measures will give reasonable protection against further decay.

The outline of the cavity being established with all margins located at a self-cleansing point, meeting the requirements of extension for prevention, we now take a small inverted cone bur about eight-tenths of a millimeter in diameter, one of the two smallest sizes made, and holding the handpiece parallel with the long axis of the tooth, with a swaying motion flatten the dentin of the seat or cervical wall, extending it well beneath the labio and linguo-cervical curves of enamel; at the same time the axio-cervical angle is squared out. This makes a substantial seat and gives ample retention to the labial and lingual at the cervical. In the dentin of each of these point angles the flat end of the bur is made to cut root wise to about the depth of the head of the bur (not more) for retainers in which to start the gold, and from the bottom of these the bur is drawn incisally along the axial wall close to the labial and lingual enamel, making the grooves, these being quite deep under the cervical third of the cavity, becoming quite shallow, about the depth of the diameter of the bur, at the center of the length of the axial wall, at which point they again gradually deepen as they approach the incisal retention. At this point an undercut is made parallel with the cutting edge (about a millimeter and a half, mesio-distally) and as near to it as the dentin will permit. The engine may now be dispensed with.

These grooves are now squared out in their entire length with hoe 6–2–6 for the cervical third, cutting root-wise, and hoe 6–2–23 for the incisal two-thirds, drawing from the cervical to the incisal. If these instruments are sharp, two or three strokes will be sufficient.

The incisal retention is now squared out parallel with the cutting edge, using hatchet 6–2–23 with a sweeping motion labio-lingually. This retention is now re-enforced by sloping the axial wall in its incisal third to the bottom of the incisal retainer.

The lingual enamel wall is now cut away in the incisal third, leaving the labial exposed from the lingual to the extent of about eight-tenths of a millimeter; this forms a definite angle extending from the incisal retainer to the cutting edge.

The remaining labial plate is beveled at the incisal to allow the gold to be built upon it for protection against incisal force. The lingual half of the axial enamel is also beveled to a corresponding extent distally at the incisal to re-enforce the enamel at this point.

The entire enamel wall is now carefully examined for imperfections, any whitish spots or unsupported enamel must be removed. The finished walls should now be parallel with the direction of the enamel rods and have a clear, vitreous appearance.

The enamel wall is now slightly beveled all around, the cavity margin including about one quarter the thickness of the enamel. All debris is carefully removed and the cavity is ready to fill.

DISCUSSION.

Dr. Harper (after reading paper): If I have not made myself clear in this presentation, or to help in any portion of the discussion, I will be glad to answer any question that may suggest itself to any one.

QUESTION: Do you do all the beveling with the chisel?

ANSWER: Yes

QUES.: Do you make this preparation universal—that is, independent of the size of the cavity in every tooth?

ANS.: I speak only of mesio or disto-incisal cavities, not simple, easy cavities.

QUES.: You speak of having some dentin next to enamel. Suppose you do not have it, do you cut away all enamel until you reach supporting dentin?

ANS: No; if all were gone I could not get any. In the incisal third I always cut away unsupported enamel, but in the cervical two-thirds you can leave unsupported enamel. I have here two specimens which I will be pleased to show which demonstrate this idea of filling. One is a model of a cavity prepared after this manner, and the other a filling of brass, which will give you an idea of the exact shape of such a filling. They are so cut out as to show the undercut at the incisor and cervical walls, and you will see that there is no real undercut except at the incisal. This is the tooth and filling. The tooth can not slide below because of substantial undercuts as shown by projections on the filling model. The filling must rotate or tip out; there is absolutely no movement; if the filling comes out it must tip out. I want to show you that there is not only resistance to the tipping strain at the incisal portion of fillings, but also half way down the tooth in this square groove, which can be seen in this case, because the filling at the tip of the incisal is uncovered.

QUES.: What do you do in those teeth in which the frail, delicate enamel almost comes together at incisal edge? I have filled lots of teeth and not left any dentin between the plates at incisal edge of the tooth. Some teeth are too thin to make it possible to have the enamel supported by dentin.

ANS: If there has been any secondary decay, or the dentin has been cut away, you can occasionally leave the enamel at the incisal edge unsupported, but still there is some risk involved in doing so. If there is no dentin between the two plates at the incisal it is an unsafe procedure to make a contour filling; you had better extend the cavity until you get enamel supported by dentin. It may be argued that this is a good method in some teeth; I say it is good in all teeth. I carry out this method in thick teeth, labio-lingually, and I find it equally good in thin teeth. I do not make the statement that you never have failures in thin teeth labio-lingually; we make failures with any method. but to my mind this is the best method.

QUES: In a case of that kind, instead of cutting away so much from incisal, why can't you save enamel at incisal edge and anchor the filling from the cervical portion of the cavity? For example: In order to get support from the dentin, why not cut away more from the labial portion of the tooth and expose more filling if you think it is so necessary to have dentin for support? Can't you get all the support you need from the cervical portion of cavity?

ANS.: The trouble is that the support at the cervical is so fär away from the point at which the force is exerted. I have seen so many bites tested; I have seen one individual who could bite with his incisors equal to a pressure of 200 pounds

QUES.: Did he travel with a circus?

ANS.: No; he was a student of dentistry, and there are lots of them. You do not need any support at the cervical. Where you anticipate any great force of masticating it can easily be determined by an examination of the teeth. I can estimate within ten pounds the amount of force a patient can exert with his jaws.

QUES.: The more you cut away from the cutting edge the more surface you are exposing, and I think pressure brought to bear upon that by mastication would break the tooth.

Ans.: There is not much danger, however. This portion of the labial and lingual wall is in mesial and distal incisal; if the cavity should include one-third of the tooth it should be carried to the nearest developmental groove. All grooves are lines of weakness, and if you cut the labial wall approximately to that groove and leave enamel it will be so weak that it will be apt to cleave. So it is my practice always, where the angle is gone, to carry the labial and lingual wall to the nearest developmental groove. It usually involves one-third of labial surface. This is a very important thing to know; in examining the developmental grooves under the microscope this fact is impressed upon you.

Ques.: How would you modify this formation if the cavity extends a little further over to the median line of the tooth?

Ans.: I would carry out the same kind of preparation. I would cut a step to carry it out to that point. I would use a little step to avoid leaving weak enamel at the incisal. It is not for retention, but simply to protect cutting edge.

Ques: This would not show at labial surface at all?

Ans.: No.

Ques.: Can you condense into a groove made with inverted cone as well as one made by round bur?

Ans.: Yes, sir. I was very much interested in that discussion last night in reference to soft foil, but I was so small that the chairman could not see me.

Ques: How would you fill that tooth with soft gold?

Ans.: I would not fill it with soft gold.

Dr. Siddall: The discussion has practically been taken out of this paper by the questions which have been asked involving the different cavities which present themselves. The principle which the essayist advances in the preparation of this cavity is much the same as that used in my practice ; that is, to get square seat. There are cases where we have extreme thinness, and in these cases the only place where I would differ would be to cut the step at the incisal edge, and not, as he said, bring it to the developmental groove on both labial and lingual, and, if decay extend further, carry it across further ; but I would bring it across and anchor on the lingual. If there is no dentin in the tooth at that point I carry it down a little further in incisal angle, provided there is plenty of dentin, but there are teeth in which this is not practicable. It seems to me the discussion upon the paper is just a question of choice between two theories: Are we going to keep round grooves, or is there any advantage in the square? I agree with essayist that square grooves are the best. There are men who believe in the round, and I will leave them to discuss this question. I may have been unfortunate in the teeth that have come under my observation, but I do not believe any great percentage of teeth are filled and dentin left between those thin plates on the incisal edge of tooth ; at least I have not filled them.

Dr. Root: I do not like the step preparation. The experience I have had is that it will break off. I believe in showing a little more gold on the labial. I used to extend over on lingual, allowing as little gold to show as possible, but now I extend over on labial and do not use the step.

Dr. Sweetnam: I would like to ask the writer of this paper, before he closes discussion, whether he leaves the filling just a little bit shorter, or whether he has the filling flush with the edge of cavity on incisal edge.

Dr. Crouse: Teeth which are decayed so much as to necessitate extending to a considerable distance over edge of the tooth beyond the developmental groove. I would carry filling over cutting edge and use No. 60 platinized gold.

Dr. Dorrance: I would like to talk an hour upon this subject, but I do not believe I will. It is important that this subject should be discussed, but at the same time I recognize the fact that we have not much time to do it. It is a well-known fact that a square line or angle is always the weak spot in any structure; at the same time I recognize that the proper condensation of gold is obtained in these cases without the strain caused by spreading of gold; therefore I entirely agree with the essayist. The point made by Dr. Crouse, that he would carry the filling over the cutting edge, my experience and observation has been that a filling made in this manner must be very thick; even platinized gold will force the plates of enamel apart.

Dr. Hoff: The objection I have to this preparation of cavity is this: In cutting of these grooves with square angles, as Dr. Dorrance has already stated, you threaten the structure of the tooth. It doubtless secures better anchorage for the filling but at the sacrifice of tooth substance, and that is one objection to this sort of preparation, it subjects enamel to a strain in many cases which it will not stand. In reference to the objections that Dr. Harper makes about filling round grooves, I fill the grooves lengthwise or axially, and not crosswise. It is easier to fill a round hole than a square one. We can condense the gold into the round groove a great deal easier than into the sharp angles.

The illustration used by Dr. Harper does not fairly represent the conditions of a properly prepared cavity. He illustrates an attempt to fill a single shallow groove by condensing the gold laterally, when the fact is that we have two grooves connected by the body of the filling making a dovetailed groove into which it is easy to pack the gold.

Another objection: If you cut the enamel until the dentin supports it you leave such a broad surface to the border of cavity that when you come to adapt the gold to it the gold will rebound and leave the border and we do not get a close joint. We can relieve this to some extent by building up from the bottom of the cavity, contouring it out, but it is difficult to adapt the gold closely to such a large flat surface.

Dr. Wood: I would like to say one word in defence of the angle. I have not had the experience of Dr. Hoff or some of the gentlemen who have spoken, but this idea struck me a year or two ago, however I have not presented it before. The objection to the angle is that it weakens the tooth. If you can have an angle one-third the depth of a curved undercut, why does it weaken the tooth any more than your round groove which is just as deep?

Dr. Hoff: It breaks at the angle, when it would not in a rounded corner.

Dr. Wood: The angular groove is so slight, only one-third the depth of bur, that it does not weaken the tooth any more than the round groove. I make my undercuts in a great many cases with the Rose bur and square with a chisel.

Dr. Dorrance: The point I wanted to make in regard to the angle is this: It is well known by all structural manufacturers that a sharp angle is certainly a p int of weakness in any structure. Now, whether it is of enough importance to take into consideration here is another matter. It is the only point in connecti n with the operation that is to my mind weak. The shape of the cavity, the material resistance of same are points which are worth noticing, but the angle is certainly the element of weakness.

Dr. Harper: The preparation of this cavity as explained has been developed, not from any theoretical con sideration of the subject, but the necessity for greater security in the retention of these fillings, and has led me to this method of preparation.

Where are the failures? Just keep account of them, examine them closely as they come under your observation. It is not the angle breaking down where we tip it out but it is in the incisal third and because of the lack of proper resistance to the tipping strain. You must get the maximum strain further down on the labial and lingual. It is said that a tooth is not as strong where we use square grooves as with round. I will concede that, but that is not what causes these failures; they result from lack of security in this incisal retention which is re enforced in this method of preparation by shallow, square grooves. They can be one-fourth or one half the depth of the round grooves and still be ten times as strong. There is no retention of the gold by a round groove. I would suggest that any one who is interested in this subject can be convinced by preparing and filling two similar cavities, one with square and the other round grooves, and then break the teeth and examine with a magnifying glass.

---

The fourth session was called to order at 9:20 A.M., Wednesday, June 13, 1900.

The president called upon Drs. Fowler and Diack to read their papers, and stated that as the papers were on similar subjects they could be discussed conjointly.

